# Scientific Validation of *Gentiana kurroo* Royle for Anti-Inflammatory and Immunomodulatory Potential

**DOI:** 10.1155/2014/701765

**Published:** 2014-02-23

**Authors:** Khan Mubashir, Khalid Ghazanfar, Bashir A. Ganai, Seema Akbar, Akhtar H. Malik, Akbar Masood

**Affiliations:** ^1^Department of Biochemistry, University of Kashmir, Srinagar 190 006, India; ^2^Regional Research Institute of Unani Medicine, Kashmir University Campus, Srinagar 190 006, India; ^3^Centre for Biodiversity and Taxonomy (CBT), Department of Botany, University of Kashmir, Srinagar 190 006, India

## Abstract

*Gentiana kurroo* Royle is a critically endangered medicinal plant species endemic to the northwestern Himalayas. This plant was studied for the immunomodulatory and anti-inflammatory potential. Carrageenan paw edema model was used to study the potential of the drug in inflammation in Wistar rats. SRBC specific haemagglutination titre and DTH assays were carried out in Balb/C mice for observing the effect of test drugs on immune system. The plant extracts were found to be active against inflammation. The methanolic fraction was observed to be the most effective in inhibition of paw edema with the inhibitory potential of 47.62%. In immunomodulation studies the plant extracts showed the immunosuppressant activity. Methanolic fraction was observed to have maximum potential for the suppression of both humoral (57.57% and 54.05%) and cell mediated immunity (65.27% and 75%). From these studies, it can be concluded that the extracts of plant are having anti-inflammatory and immunosuppressant activity. Since in chronic inflammation like arthritis there is the involvement of immune system, this plant may serve as an alternative for the treatment of autoimmune diseases like arthritis.

## 1. Introduction

A number of medicinal herbs have been found to be useful for pharmacological activities, like anti-inflammatory and immunosuppressive effects. Throughout the world different types of inflammatory diseases like rheumatic diseases are very common [1]. A major cause in development of inflammatory and autoimmune diseases is alteration in the T-cell responses and irregular functioning of the immune system [2]. Any changes in the immune response involving induction, expression, amplification, or inhibition of any part or phase of the immune response refer to immunomodulation. The agents that can modulate the immune response are traditionally called the immunomodulators and are stimulatory or suppressive in their effect [3].

The immune system can be modulated by a large number of immunosuppressive drugs that have different mechanisms of action. Most common of these drugs are the glucocorticoids used widely in inflammatory disease management. These drugs by affecting gene transcription events cause inhibition of various immune functions [4, 5]. An alternative to conventional chemotherapy is the use of herbal products for immune suppression and there is an increasing demand to discover new drugs with fewer side effects. The plant products which have been found to be able to affect the functioning of the immune system are mainly the primary and secondary metabolites [6]. Such compounds with the capacity to inhibit cellular and humoral immune responses can have useful applications in some immune-mediated disorders including autoimmune diseases [7]. Because of the side effects associated with the synthetic drugs and increased demand of herbal medicines for therapeutic applications, it has become necessary to exploit the medicinal potential of plant species.

*Gentiana kurroo* Royle belongs to the family Gentianaceae and is a critically endangered (CR) medicinal plant species, endemic to the northwestern Himalayas. The drug (rootstock) of this plant is administered in fevers and urinary complaints, also used as a bitter tonic, antiperiodic, expectorant, antibilious, astringent, stomachic, antihelminthic, blood purifier, and carminative [8]. The methanolic root extract of this plant contains tannins, alkaloids, saponins, cardiac glycosides, terpenes, flavonoids, phenolics, and carbohydrates. The root extract of this plant has been found to have an analgesic activity [9]. The ethanolic extract of the flower tops of this plant contains alkaloids, flavonoids, glycosides, free phenols, and sterols/terpenes and thus has been found to show an anti-inflammatory activity [10].

The present study was carried out to evaluate the role of different fractions of *G. kurroo* in inflammation and immunomodulation.

## 2. Materials and Methods

### 2.1. Collection and Identification of Plant Material

The plant material was procured from subalpine region in Dachigam and identified in the Centre of Plant Taxonomy (COPT), Department of Botany, University of Kashmir. The specimen is retained in the herbarium of COPT vide voucher number 1804-KASH.

### 2.2. Preparation of Extracts

The whole plant was washed, cut into small pieces, and shade-dried. The plant material was pulverized into coarse powder and extracted successively using petroleum ether, ethyl acetate, methanol, and water, respectively, by soxhlet extraction. The solvents were allowed to evaporate in a rotary evaporator at 40°–45°C and the extracts obtained were stored in a refrigerator at 4°C. The yields of the petroleum ether, ethyl acetate, methanol, and aqueous extracts were 5.6, 4.3, 5.3, and 4.2% (w/w), respectively.

### 2.3. Animals

Male albino Wistar rats (120–140 gm) in groups of 4 each were used for anti-inflammatory study. Drugs were prepared in 1% tween 20 and administered orally at doses of 250 mg/kg bw. Diclofenac was used as an anti-inflammatory drug at a dose of 20 mg/kg (p.o).

Male Balb/C mice, 8–10 weeks old and weighing 18–22 g, in groups of five each, were used for the immunomodulatory study. Drugs for oral administration were freshly prepared as a homogenised suspension of different extracts of *G. kurroo* at doses of 100 mg/kg each in 1% tween 20 and administered orally, once daily for the duration of the experiment to Balb/C mice. Cyclophosphamide was used as the standard immunosuppressive agent at 50 mg/kg (p.o).

The animals were housed under standard laboratory conditions with a temperature of (25 ± 2)°C, relative humidity of (55 ± 10)%, and 12/12 h light-dark cycles and fed with standard pellet diet (Lipton India Ltd.) and water was given *ad libitum*. None of the animals was sacrificed throughout the study.

### 2.4. Chemicals

Tween 20, cyclophosphamide, and diclofenac were purchased from Sigma chemical Co. (St. Louis, MO). All other reagents used were of analytical grade.

Fresh blood was collected from healthy sheep in the animal house. Sheep red blood cells (SRBCs) were washed thrice with normal saline adjusted to a concentration of 1 mL, containing 5 × 10^9^ cells for immunisation and challenge.

### 2.5. Experimental Protocols

All experimental protocols and the number of animals used for the experimental work were duly approved by the Institutional Animals Ethics Committee (IAEC) of Indian Institute of Integrative Medicine (CSIR), Canal Road Jammu (CPCSEA registration no. 67/CPCSEA/99).

#### 2.5.1. Carrageenan-Induced Paw Edema

Carrageenan-induced paw edema model [11] was utilized to assess the acute anti-inflammatory potential of the test samples. Animals were divided into six groups (*n* = 4). Group I served as control, rats in groups II–V were administered with plant extracts, and group VI was used as positive control. All drugs were given orally 45 min before Carrageenan injection. Carrageenan was prepared in normal saline (1%) and 0.1 mL was injected into the subplantar region of left hind paw. The volume of both paws was measured with volume differential meter (520-R, IITC Life Science, USA) after 4 hrs with the volume of right paw taken as uninjected paw volume. Percent of inhibition was calculated by taking mean of the difference of right and left paw edema, using the formula

(1) $$\begin{array}{c} \%\;\;\text{inhibition}=\frac{C-T}{C}\times 100, \end{array}$$

where  *C* represents mean edema in the control group and *T* represents mean edema in the treated group.

#### 2.5.2. Humoral Antibody Response

The mice were divided into 6 groups, each consisting of 5 animals. Mice in group I (control) were given 1% tween 20, 0.2 mL/mice for 14 days. Mice in groups II–V were given a drug dose of 100 mg/kg bw (orally) for 14 days. Mice in group VI were given cyclophosphamide 50 mg/kg on day 1 and continued for 14 days. The animals were immunised by injecting 200 *μ*L of 5 × 10^9^ SRBCs/mL, intraperitoneally (i.p) on day 1. Blood samples were collected in microliter tubes from individual animals of all the groups by retroorbital vein puncture on day 7 and day 14. The blood samples were centrifuged and the serum was separated. Then haemoglutination primary and secondary titres were performed [12, 13].

#### 2.5.3. Delayed-Type Hypersensitivity

This method was carried out to determine the effect of extracts on the cell-mediated immunity. Animals were divided into six groups of 5 each. Group I served as sensitized control, as in humoral antibody titre. Mice in groups II–V were administered extracts of *G. kurroo* after SRBC's sensitization and once daily for seven days. Cyclophosphamide (50 mg/kg) was administered as standard T-cell suppressor (group VI). The mice were challenged by injecting the same amount of SRBCs subcutaneously into the right hind footpad, whereas left hind footpad served as control [14, 15]. The footpad thickness was measured with micrometre calliper (pitch 0.01 mm) at 24 and 48 h of SRBC's challenge.

### 2.6. Statistical Analysis

Data were expressed as mean ± S.E.M and statistical analysis was carried out employing the ANOVA followed by Tukey's HSD test using SPSS 16 software.

## 3. Results

### 3.1. Carrageenan-Induced Paw Edema

Different plant extracts used in the current study were found to show an anti-inflammatory activity. As compared to control group the treated groups showed decrease in the edema formation after 4 hrs. Ethyl acetate and aqueous fractions produced an inhibition of 23.81% and 28.57%, respectively, in the paw edema. Petroleum ether fraction was found to have the minimum inhibition in edema (7.62%). The maximum inhibition (47.62%) in the edema formation was obtained in the methanolic fraction at a dose of 250 mg/kg bw (Table 1). Diclofenac was used as a standard anti-inflammatory drug at a dose of 20 mg/kg bw. The results obtained in the methanolic fraction were found to be significantly (*P* < 0.05) related to diclofenac treated group (55.24%).

### 3.2. Haemagglutination Antibody Titre

**Figure 1:**
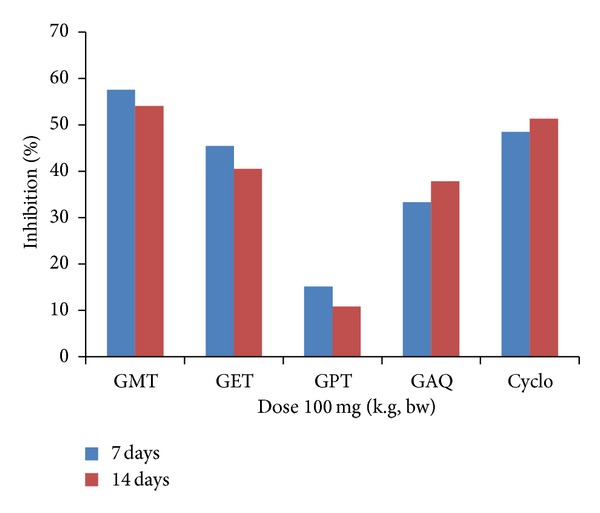
Graph showing % age inhibition of primary and secondary response in Balb/C mice by different fractions of *Gentiana kurroo* Royle.

The antibody titre was carried out to evaluate the influence of extracts on the production of antibodies by B-cells. The extracts were administered to the mice at a dose of 100 mg/kg bw and were seen to decrease the primary as well as the secondary immune response (Table 2). Methanolic extract showed marked decrease in the HA titre as compared to control with the percentage of age inhibition of 57.57% and 54.05% in the primary and secondary response, respectively (Figure 1). The other extracts showed less effect on humoral response with the least effect observed in petroleum ether extract. The results obtained in the cyclophosphamide treated group were found to be insignificant (*P* < 0.05) in relation with the methanolic extract treated group and control group.

### 3.3. Delayed-Type Hypersensitivity

**Figure 2:**
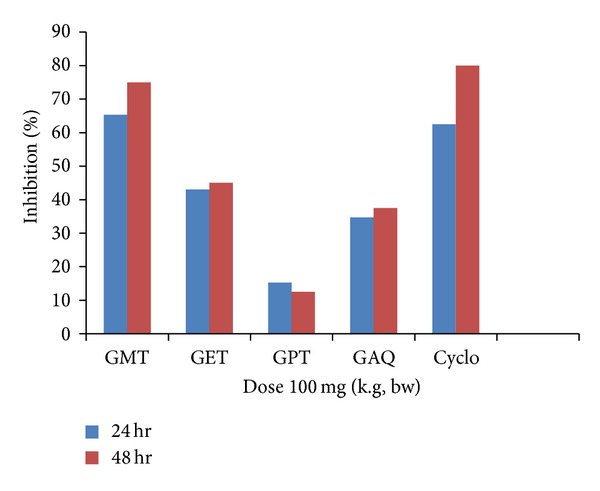
Graph showing 24 h and 48 h % age inhibition of cell mediated response in mice by different fractions of *Gentiana kurroo* Royle.

DTH was carried out to study the effect of the different plant extracts on the nonspecific cellular immune response. All the plant extracts tested were found to decrease the cellular immune response (Table 3). The effect on edema formation was studied after 24 h and 48 h with the extracts fed to the mice at a dose of 100 mg/kg bw. The results obtained in the methanolic extract treated group significantly (*P* < 0.05) showed the inhibition of 65.27% and 75% of edema formation after 24 h and 48 h, respectively (Figure 2). Petroleum extract treated group was observed to have minimum suppressive effect on cellular immune response with inhibition of 15.27% and 12.5% after 24 h and 48 h. In the standard group treated with cyclophosphamide at a dose of 50 mg/kg, the percentage of age inhibition obtained was 62.5% and 80% as compared to control group. The results obtained were found to be significant in relation with control group at *P* < 0.05.

## 4. Discussion

Inflammation is a protective process that is essential for the preservation of the integrity of the organism in the event of physical, chemical, and infectious damage. Often, it is found that inflammatory response to severe lesions erroneously damages normal tissue [16]. Analysis of the results in the present study indicated that the different extracts of *G. kurroo* have anti-inflammatory potential. Of these different extracts methanolic fraction was found to have the maximum activity. This may be because of the presence of more bioactive agent(s) in this fraction. Most of the bioactive agents present in the medicinal herbs belong to secondary metabolites. These may be terpenoids or flavonoids in nature. As many monoterpenoids like camphene, borneol, and *β*-pinene are known to possess an anti-inflammatory property [17, 18], flavonoids like 6-methoxytricin have been seen to show anti-inflammatory and analgesic activities [19]. During acute inflammation leukocytes and serum proteins migrate to areas of tissue injury. Recruitment of cells to inflammatory sites is dependent on the release of vasoactive and chemotactic factors that increase regional blood flow and microvascular permeability and promote the migration of leukocytes from the intravascular space into the tissues [20]. It is well established that activated immunocytes are involved in the inflammation process, particularly macrophages, which play a crucial role in the specific and nonspecific immune responses during inflammation [21]. Further these fractions were used to demonstrate the effect on the immune system using Balb/C model for the study. All the fractions were again observed to have an immunosuppressive effect by inhibiting the antibody formation and the cellular immune response. Methanolic fraction was again found to have more inhibitory effect on the immune response. No such earlier study is found to be carried out on this plant. The results of the present study show that *G. kurroo* possesses an anti-inflammatory potential and this may be because of the decrease in both the specific and nonspecific immune response.

## 5. Conclusion

*G. kurroo* is a medicinal herb endemic to northwestern Himalayas. This plant is locally used for the treatment of many diseases. The current study was undertaken to explore the folklore claim of this plant. From the results demonstrated above, it seems that the plant may be an important reservoir of natural drugs. The results suggest that the plant can be a useful drug resource for the treatment of inflammatory diseases and several immunostimulant clinical conditions. Further studies are needed for the isolation and identification of the bioactive components in the plant.

## Figures and Tables

**Table 1:** Effect of different extracts of *Gentiana kurroo *Royle at a dose of 250 mg/kg on Carrageenan-induced paw edema in rats (mean ± S.E) (*n* = 4).

Serial number	Groups	Dose (mg/kg)	Initial paw volume (mL)	Paw volume after 4 h (mL)	Edema (4 h)	% age inhibition (4 h)
1	Control	NS	2.07 ± 0.075	1.02 ± 0.048	1.05 ± 0.029^a^	—
2	GMT	250	1.55 ± 0.029	1 ± 0.041	0.55 ± 0.029^b^	47.62
3	GET	250	1.8 ± 0.071	1 ± 0.041	0.8 ± 0.071^ac^	23.81
4	GPT	250	1.95 ± 0.056	0.97 ± 0.041	0.97 ± 0.074^bc^	7.62
5	GAQ	250	1.72 ± 0.025	0.97 ± 0.025	0.75 ± 0.029^b^	28.57
6	Diclo	20	1.57 ± 0.025	1.1 ± 0.041	0.47 ± 0.025^c^	55.24

Values along the same column with different superscripts are statistically significant to each other using Tukey's HSD test (*P* < 0.05). GMT: methanolic extract; GET: ethyl acetate extract; GPT: petroleum ether extract; GAQ: aqueous extract; Diclo: diclofenac.

**Table 2:** Effect of different extracts of *Gentiana kurroo *Royle on SRBC specific humoral immune response in Balb/C mice. (mean ± S.E) (*n* = 5).

Humoral response			
Groups	Dose (mg/kg)	Primary titre	Secondary titre
Control	SRBC	6.6 ± 0.24^bc^	7.4 ± 0.24^a^
Methanolic fraction	100	2.8 ± 0.22^c^	3.4 ± 0.24^c^
Ethyl acetate fraction	100	3.6 ± 0.27^bc^	4.4 ± 0.24^bc^
Petroleum ether fraction	100	5.6 ± 0.24^a^	6.6 ± 0.24^a^
Aqueous fraction	100	4.4 ± 0.24^b^	4.6 ± 0.24^b^
Cyclophosphamide	50	3.4 ± 0.24^bc^	3.6 ± 0.24^bc^

Values along the same column with different superscripts are statistically significant to each other using Tukey's HSD test (*P* < 0.05).

**Table 3:** Effect of different extracts of *Gentiana kurroo *Royle on SRBC specific cellular immune response in Balb/C mice (mean ± S.E) (*n* = 5).

DTH assay			
Groups	Dose (mg/kg)	24 hr paw thickness (mm)	48 hr paw thickness (mm)
Control	SRBC	0.72 ± 0.016^a^	0.4 ± 0.027^a^
Methanolic fraction	100	0.25 ± 0.021^d^	0.1 ± 0.013^d^
Ethyl acetate fraction	100	0.41 ± 0.019^c^	0.22 ± 0.032^c^
Petroleum ether fraction	100	0.61 ± 0.035^b^	0.35 ± 0.04^ab^
Aqueous fraction	100	0.47 ± 0.016^c^	0.25 ± 0.029^bc^
Cyclophosphamide	50	0.27 ± 0.009^d^	0.08 ± 0.007^d^

Values along the same column with different superscripts are statistically significant to each other using Tukey's HSD test (*P* < 0.05).
